# Supplementary material for: EnzML: multi-label prediction of enzyme classes using InterPro signatures
Source: BMC Bioinformatics. 2012 Apr 25;13:61. doi: 10.1186/1471-2105-13-61 (PMC3483700; doi:10.1186/1471-2105-13-61)
Supplement: Addtional file 5 — The Java code to format the data files, evaluate and predict. The file enzml_java_code.tar.gz contains the Java code used to format database data to ARFF and XML formats, to execute cross and train-test (jackknife) evaluations and to record evaluation results to database. More information is included in the readme.txt file and the Javadoc files. The code can be used with a MySQL database. To use a different database software, other JDBC drivers might be required. [file 1471-2105-13-61-S5.gz › java_code/enzml2011/doc/uk/ac/ed/inf/csb/taweka/SparseInstanceWithId.html]

SparseInstanceWithId


---


|  |  |  |
| --- | --- | --- |
| **Package** \| **Class** \| **Use** \| **Tree** \| **Deprecated** \| **Index** \| **Help** | |  |
| **PREV CLASS**   **NEXT CLASS** | **FRAMES**    **NO FRAMES**     **All Classes** |  |
| SUMMARY: NESTED \| FIELD \| CONSTR \| METHOD | DETAIL: FIELD \| CONSTR \| METHOD |  |


---


## uk.ac.ed.inf.csb.taweka Class SparseInstanceWithId

```
java.lang.Object
  weka.core.Instance
      weka.core.SparseInstance
          uk.ac.ed.inf.csb.taweka.SparseInstanceWithId
```

**All Implemented Interfaces:**: java.io.Serializable, weka.core.Copyable, weka.core.RevisionHandler

---

``` public class SparseInstanceWithId extends weka.core.SparseInstance ```

A Weka SparseInstance with an identifier,
to make it easier to retrieve a certain instance (row) in the ARFF file.
For biological applications, the identifier can be the protein or gene identifier from a public database.
The identifier makes it easier to retrieve the row (or prediction) for a given protein/gene.

**Version:**
:   27 Apr 2010

**Author:**
:   Luna De Ferrari luna.deferrari-at-ed.ac.uk

**See Also:**: Serialized Form

---

| **Field Summary** | |
| --- | --- |
| `java.lang.String` | `m_id`             The instance identifier |


**Constructor Summary**

`SparseInstanceWithId()`


| **Method Summary** | |
| --- | --- |
| `java.lang.String` | `getId()` |
| `java.lang.String` | `toString()` |

**Methods inherited from class weka.core.SparseInstance**

`attributeSparse, copy, getRevision, index, isMissing, locateIndex, main, mergeInstance, numAttributes, numValues, replaceMissingValues, setValue, setValueSparse, toDoubleArray, value`

**Methods inherited from class weka.core.Instance**

`attribute, classAttribute, classIndex, classIsMissing, classValue, dataset, deleteAttributeAt, enumerateAttributes, equalHeaders, hasMissingValue, insertAttributeAt, isMissing, isMissingSparse, isMissingValue, missingValue, numClasses, relationalValue, relationalValue, setClassMissing, setClassValue, setClassValue, setDataset, setMissing, setMissing, setValue, setValue, setValue, setWeight, stringValue, stringValue, toString, toString, value, valueSparse, weight`

**Methods inherited from class java.lang.Object**

`equals, getClass, hashCode, notify, notifyAll, wait, wait, wait`

**Field Detail**

### m\_id

```
public java.lang.String m_id
```

:   The instance identifier


**Constructor Detail**

### SparseInstanceWithId

```
public SparseInstanceWithId()
```


**Method Detail**

### toString

```
public java.lang.String toString()
```

:   **Overrides:**: `toString` in class `weka.core.SparseInstance`

---


### getId

```
public java.lang.String getId()
```


---


|  |  |  |
| --- | --- | --- |
| **Package** \| **Class** \| **Use** \| **Tree** \| **Deprecated** \| **Index** \| **Help** | |  |
| **PREV CLASS**   **NEXT CLASS** | **FRAMES**    **NO FRAMES**     **All Classes** |  |
| SUMMARY: NESTED \| FIELD \| CONSTR \| METHOD | DETAIL: FIELD \| CONSTR \| METHOD |  |


---
